# Overcoming radio-resistance in esophageal squamous cell carcinoma via hypermethylation of PIK3C3 promoter region mediated by KDM5B loss

**DOI:** 10.1093/jrr/rrac004

**Published:** 2022-03-25

**Authors:** Xiaobo Wang, Min Gu, Yongjian Ju, Juying Zhou

**Affiliations:** Department of Radiation Oncology, The First Affiliated Hospital of Soochow University, Suzhou 215006, Jiangsu, P.R. China; Department of Radiation Oncology, The First People’s Hospital of Nantong, Nantong 226001, Jiangsu, P.R. China; Department of Radiation Oncology, The First People’s Hospital of Nantong, Nantong 226001, Jiangsu, P.R. China; Department of Radiation Oncology, The First People’s Hospital of Nantong, Nantong 226001, Jiangsu, P.R. China; Department of Radiation Oncology, The First Affiliated Hospital of Soochow University, Suzhou 215006, Jiangsu, P.R. China

**Keywords:** esophageal squamous cell carcinoma (ESCC), KDM5B, PIK3C3, radiotherapy, autophagy

## Abstract

Many patients with esophageal squamous cell carcinoma (ESCC) are inoperable because of old age or the advanced stage of the disease; thus radio- and chemotherapy are believed as the standard treatments for these patients. However, due to the radio-resistance of tumor cells that may develop during radiotherapy, results remain unsatisfactory. In this article, the possible relationship between the expression of lysine demethylase 5B (KDM5B) and ESCC radio-resistance is clarified, and the underlying mechanism is evaluated. Using the GSE75241 microarray, we identified KDM5B as a potential oncogene in ESCC. KDM5B was overexpressed in ESCC patients and cells. Inhibition of KDM5B enhanced the H3K4me3 methylation of phosphatidylinositol 3-kinase catalytic subunit type 3 (PIK3C3) promoter and induced the expression of PIK3C3. Knockdown of KDM5B or overexpression of PIK3C3 in KYSE-150 and TE-10 cells promoted apoptosis, cell cycle arrest, autophagy, and increased sensitivity to radiotherapy. Silencing of PIK3C3 attenuated the promoting effect of sh-KDM5B on the sensitivity of ESCC cells to radiotherapy. The inhibition of sh-KDM5B in radio-resistance of ESCC cells was also reproduced *in vivo*. Taken together, our findings provide evidence that reduced expression of KDM5B has a critical role in promoting ESCC radio-sensitivity by upregulating PIK3C3, suggesting KDM5B may function as an oncogene in ESCC.

## INTRODUCTION

Esophageal cancer ranks seventh in terms of incidence (572 000 new diagnoses) and sixth in overall mortality (509 000 deaths), signifying that it will be responsible for an estimated one in every 20 cancer deaths in 2018 worldwide [1]. Esophageal squamous cell carcinoma (ESCC) makes up for about 90% of the new esophageal cancer diagnoses each year, and regions of high incidences include Eastern to Central Asia [2]. Alcohol consumption and cigarette smoking have been identified as major contributing risk factors for ESCC [3]. Radiotherapy is a curative treatment modality for ESCC patients, and the 5-year survival rates of patients after radiotherapy is less than 20% because of radio-resistance [4]. Hence, the presence of this challenges in the treatment of ESCC means that there is an urgent need for elucidating molecular mechanism behind ESCC radio-resistance.

The phosphoinositide 3-kinase (PI3K) family is vital to almost all aspects of cell and tissue biologies and essential to human cancers and aging, and there is a recognition of the significance of distinct roles for each of the three PI3K classes (I, II and III), as well as for the different isoforms within each class [5]. PIK3 catalytic subunit type 3 (PIK3C3, also designated as VPS34) specifically generates PtdIns3P, which recruits proteins containing FYVE or PX domains, thereby originating different complexes at the membranes of endosomes, phagosomes and autophagosomes [6]. Our published data revealed that PIK3C3 was poorly expressed in ESCC tissues and predicted a dismal prognosis in patients, and PIK3C3 overexpression sensitized KYSE-150 and TE-12 cells to irradiation [7]. Meanwhile, exposure to radiation causes cellular stress, directly or indirectly by the production of reactive oxygen species, DNA damage, and autophagy [8]. However, whether PIK3C3 is involved in the radio-resistance in ESCC through the mediation of autophagy is virtually unexplored. Epigenomic alterations, such as alterations in DNA methylation, histone modification, and RNA editing, have been observed in ESCC [9]. However, it is not clear what proportion of ESCC cells carry these epigenomic changes or how they lead to tumor development. Lysine-specific demethylase 5B (KDM5B) represses the expression of genes transcriptionally by erasing the methyl group from H3K4me2/3 and serves as an oncogene and associates with human cancers closely [10]. Importantly, KDM5B overexpression predicted somber prognoses in patients with squamous cell carcinoma of the head and neck [11]. The role of KDM5B in controlling radio-resistance in cancers, especially in ESCC remains quite unknown. With these findings taken into consideration, we hypothesized the implication of the KDM5B/PIK3C3 axis in radio-resistance of ESCC.

## MATERIALS AND METHODS

### Study participants

Prior to sample collection, informed consent was acquired from all patients in accordance with the protocols approved by the institutional review boards of the First Affiliated Hospital of Soochow University. All procedures involving patients were performed following the *Declaration of Helsinki.* Thirty ESCC tissues and corresponding paracancerous esophageal epithelial tissue specimens were obtained from patients who underwent surgical resection at the First Affiliated Hospital of Soochow University from January 2019 to September 2020. The patients were aged between 58 to 76 years and included 19 male and 11 female cases. The normal paracancerous esophageal epithelial tissues were at least >3 cm from the tumor margin. The 30 samples used in this study were confirmed by experienced clinicopathologists. ESCC tissues from patients were placed in liquid nitrogen and then stored at −80°C until analysis. Patients who had received radiotherapy or chemotherapy, patients with infections, patients with a history of malignancy other than ESCC, and patients with primary immunodeficiency and acquired immunodeficiency syndrome were excluded from this study.

### Cell culture and transfection

ESCC cell lines KYSE-150 and TE-10 were from National Infrastructure of Cell Line Resource (Beijing, China). ESCC cell lines ECA-109, EC-9706 and normal esophageal epithelial cells HET-1A were acquired from Shanghai EK-Bioscience Biotechnology Co., Ltd. (Shanghai, China). The cells were preserved in RPMI-1640 medium (Gibco, Invitrogen, Carlsbad, CA, USA) with 10% FBS (Gibco), 100 U/mL penicillin and 100 mg/mL streptomycin (Beyotime Biotech, Shanghai, China). The cultures were incubated in an incubator at 37°C with 5% CO_2_, and the medium was refreshed at 1 d intervals.

Short hairpin RNA (sh)-KDM5B, sh-PIK3C3, overexpression (oe)-PIK3C3 and their corresponding negative controls were synthesized by GenePharma (Shanghai, China). Prior to transfection, the KYSE-150 and TE-10 cell were plated in 6-well plates to achieve an approximately 80% confluence. Transfection was performed using the lipofectamine 3000 transfection kit (Invitrogen) as per the manufacturer’s protocol, and the cells were screened with 2 μg/mL puromycin (Sigma-Aldrich Chemical Company, St Louis, MO, USA) to obtain stably transfected cell lines.

### RNA isolation and quantification analysis

Total RNA was isolated from ESCC tissues, normal adjacent tissues, and cells using TRIzol (Invitrogen) as per the manufacturer’s protocol. The purity and concentration of RNA were assessed by NanoDrop 2000 (Thermo Fisher Scientific Inc., Waltham, MA, USA). Then, the RNA was reverse transcribed into cDNA using a reverse transcription kit (K1621, Fermentas, Maryland, NY, USA). The primer sequences of KDM5B and PIK3C3 (Table 1) were synthesized by Sangon (Shanghai, China). The mRNA expression of each gene was detected by RT-qPCR (ABI 7500, ABI Company, Oyster Bay, NY, USA) according to the fluorescent quantitative PCR kit (TaKaRa, Dalian, Liaoning, China). The expression of each mRNA was normalized to glyceraldehyde–3-phosphate dehydrogenase (GAPDH), and the data were analyzed using the 2^-ΔΔCt^ method.

**Table 1:** Primer sequences for RT-qPCR

Name of primer	Sequences (5′-3′)
KDM5B-F	AGCCAGAGACTGGCTTCAGGAT
KDM5B-R	AGCCTGAACCTCAGCTACTAGG
PIK3C3-F	GCGTTCTTTGCTGGCTGCACAA
PIK3C3-R	CTCCAAGCAATGCCTGTAGTCTC
GAPDH-F	ACCACAGTCCATGCCATCAC
GAPDH-R	TCCACCACCCTGTTGCTGTA

**Note:** RT-qPCR, reverse transcription quantitative polymerase chain reaction; KDM5B, lysine demethylase 5B; PIK3C3, phosphatidylinositol 3-kinase catalytic subunit type 3; GAPDH, glyceraldehyde–3-phosphate dehydrogenase; F, forward; R, reverse.

### Western blot

KYSE-150, TE-10 cells and ESCC cells-formed xenograft tumor tissues were lysed with radioimmunoprecipitation assay lysis buffer (Beyotime). Protein concentrations were assessed using the bicinchoninic acid protein quantification kit (Beyotime). Equal amounts of protein (50 μg) from each sample were separated using 10% SDS-PAGE and electrophoretically transferred to polyvinylidene fluoride membranes (Millipore, Billerica, MA, USA). The membranes were closed with 5% BSA at 37°C for 60 min and treated with primary antibodies to GAPDH (1:10000, ab181602, Abcam, Cambridge, MA, USA), KDM5B (1:2000, ab181089, Abcam), PIK3C3 (1:2000, ab124905, Abcam), H3K4me3 (1:1000, ab213224, Abcam), Cyclin B1 (1:2000, GTX100911, GeneTex, Inc., Alton Pkwy Irvine, CA, USA), CDC2 (1:1000, GTX108120, GeneTex), pCDC2 (Tyr15, 1:2000, GTX128155, GeneTex), Beclin-1 (1:1000, #3495S, Cell Signaling Technologies, Beverly, MA, USA), LC-3I/II (1:1000, #12741S, CST), and Bcl-2 (1:1000, #4223S, CST) overnight at 4°C. Subsequently, the membrane was incubated with horseradish peroxidase (HRP)-coupled secondary antibody (1:5000, ab205718, Abcam) for 60 min at room temperature. Protein bands were analyzed using Pierce Western Blotting ECL substrate kit (Thermo Fisher Scientific) and BandScan 5.0 system (Bio-Rad, Hercules, CA, USA).

### Colony formation assay

The sensitivity of cells to x-rays was detected using the colony formation assay. Briefly, the KYSE-150 and TE-10 cells were plated in 96-well plates at 6 × 10^3^ cells/well and cultured overnight. The cells were radiated at 0, 2, 4, 6, 8 Gy for 24 h [7]. The cells were continued to be cultured in 6-well plates for 3 weeks, followed by a 15 min methanol fixation and a 1 h 2% crystal violet staining. Images of the colonies were captured using a ChemiDoc imaging system (Bio-Rad), and then the colonies were counted using Image J software to assess the viability of the cells.

### Flow cytometry

The transfected KYSE-150 and TE-10 cells (1 × 10^5^ per well) were plated in 6-well plates for 24 h and exposed to 0 Gy or 8 Gy X-rays for 24 h. The apoptotic cells were distinguished by dual staining with Annexin-V fluorescein isothiocyanate (FITC)-PI and analyzed by flow cytometry (BD FACS Calibur) using an apoptosis detection kit (Keygen Biotech, Nanjing, Jiangsu, China).

For cell cycle assessment, the cells were cultured and expose to 0 Gy or 8 Gy X-rays. The cells were cultured with 6 μL 1 g/L RNase A, 1 mL 1 mg/mL PI and 400 μL PBS at ambient temperature in darkness for 15 min. A flow cytometer (FCM, BD FACS Calibur) was used to assess DNA content and cell cycle distribution.

### Chromatin immunoprecipitation

Chromatin immunoprecipitation (ChIP) assays were performed using a commercial kit (Beyotime) as per the manufacturer’s protocol. KYSE-150 and TE-10 cells were fixed with 1% formaldehyde for 10 min at room temperature. Glycine (0.1 M) was added for a 5 min incubation to quench the cross-linking reaction. The cell lysates were prepared using an ice-cold cell lysis buffer at 4°C for 60 min and sonicated to break chromatin to an average length of 500 to 800 bp. The samples were cleaned with protein A agarose (Roche Diagnostics, Co., Ltd., Rotkreuz, Switzerland) by gently spinning at 4°C for 60 min and incubated with rabbit antibodies to KDM5B (1:50, #15327S, Cell Signaling Technologies) and H3K4me3 (1:100, ab213224, Abcam) overnight at 4°C. Antibodies were used for immunoprecipitation of cell lysates, and phenol/chloroform was used for extraction, purification and recovery of DNA fragments. Finally, the DNA released from the precipitated complexes was analyzed by qPCR.

### Monodansylcadaverine staining

Cellular autophagy staining assay kit (Beijing Solabio Life Sciences Co., Ltd., Beijing, China) was used to detect autophagy in ESCC cells. KYSE-150 and TE-10 cells were exposed to 0 Gy or 8 Gy x-rays for 24 h, and the cell concentration was adjusted to 10^6^/mL with 1× Wash buffer. The cell suspension (90 μL) was stained with 10 μL monodansylcadaverine (MDC) staining for 0.5 h at room temperature in the darkness. The slides were covered with 100 μL collection buffer, observed, and photographed under a fluorescent microscope (Nikon Corporation, Tokyo, Japan).

### *In vivo* tumor formation assay

Animal studies were approved by the Institutional Animal Care and Use Committee of the First Affiliated Hospital of Soochow University. Twelve BALB/c female nude mice (4 weeks old) were purchased from the Shanghai Lab Animal Research Center (Shanghai, China). To assess the effect of KDM5B on modulating radiosensitivity in tumor models, KYSE-150 cells (5 × 10^6^) transfected with sh-NC or sh-KDM5B were injected subcutaneously into the right dorsal flank of nude mice. Two weeks after inoculation, the mice were subjected to x-ray irradiation (0 or 8 Gy) 2 times a week. The tumor volume was measured once a week with calipers. The formula for calculating the tumor volume was as follows: Tumor volume = (length × width^2^)/2. The nude mice were euthanized by intraperitoneal injection of 1% pentobarbital sodium (150 mg/kg) after 4 weeks, and the tumors were weighed.

### Immunohistochemistry

ESCC tissues, paracancerous tissues and xenograft tissues were fixed in 4% paraformaldehyde, paraffin-embedded, and sectioned at 5 μm. The sections were dewaxed in xylene, rehydrated in graded ethanol, and sealed in 3% hydrogen peroxide for 15 min for endogenous peroxidase. The sections were probed with KDM5B (1:100, GTX60284, GeneTex, Inc., Alton Pkwy Irvine, CA, USA) and Ki-67 (1:400, #9027S, CST) at 4°C and re-probed with goat anti-rabbit secondary antibody (1:2000, ab205719, Abcam) coupled with HRP for 60 min at room temperature. Next, after being cultured with diaminobenzidine (Dako, Hamburg, Germany), counter-stained with hematoxylin (Sigma-Aldrich) for 1 min and sealed with neutral gum, the sections were photographed under a microscope (Leica Microsystems GmbH, Wetzlar, Germany).

### Terminal deoxynucleotidyl transferase-mediated 2’-Deoxyuridine 5′-triphosphate nick end labeling

Apoptosis was assessed in xenograft tumor tissue sections (5 μm) by Terminal deoxynucleotidyl transferase-mediated 2′-Deoxyuridine 5′-Triphosphate nick end labeling (TUNEL) using the In-Situ Cell Death Detection Kit-POD (Roche). The sections were treated with proteinase K (10 mmol/L) for 15 min and then treated with TUNEL reaction mixture for 60 min, followed by incubation in converter-peroxidase for 30 min. TUNEL-positive cells were considered apoptotic. Fluorescence images were obtained using a fluorescence microscope (Nikon Corporation). Under the microscope, the percentage of apoptotic cells was calculated as the rate of TUNEL-positive cells to total cells.

### Statistical analysis

All statistical analyses from at least three separate experiments were performed using SPSS 23.0 software (IBM Corp, Armonk, NY, USA) and GraphPad Prism 8.0.2 (GraphPad Software Inc., La Jolla, CA, USA). Measurement data were presented as mean ± standard deviation. The paired *t* test was applied for comparisons between two groups, while one-way or two-way ANOVA followed by Tukey’s post hoc test was adopted for comparison among multiple groups. Differences that produced *P* values less than 0.05 were accepted as significant.

**Fig. 1.**
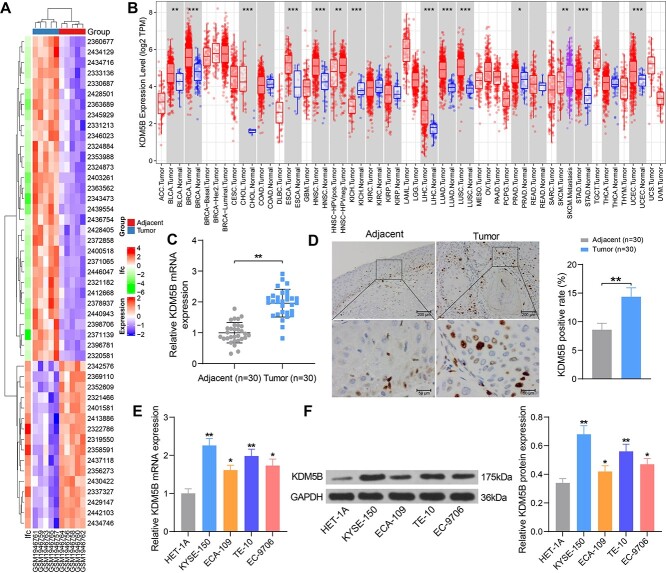
KDM5B expression is upregulated in ESCC tissues and cell lines. (A) Heatmap analysis of the top 47 significantly differentially expressed genes. (B) Analysis of KDM5B expression in multiple cancers using TIMER (https://cistrome.shinyapps.io/timer/). (C–D) Detection of KDM5B expression in ESCC tissues and normal tissues adjacent to cancer by RT-qPCR (C) and immunohistochemistry (D). (E–F) Detection of mRNA and protein expression of KDM5B in normal human esophageal epithelial cells and four ESCC cell lines by RT-qPCR (E) and Western blot (F). ^*^P < 0.05, ^*^^*^P < 0.01, ^*^^*^^*^P < 0.001 compared with adjacent tissues or HET-1A cells. The results were measurement data, which were expressed as the mean ± SD (*n* = 30). Comparisons between two groups were conducted using paired t-test (C, D), and comparisons between multiple groups analyzed by one-way ANOVA (E, F) with Tukey’s post hoc test. The experiment was independently repeated three times.

## RESULTS

### KDM5B is highly expressed in ESCC

We obtained GSE75241 from the GEO database (https://www.ncbi.nlm.nih.gov/geo/) with a data series consisting of 4 ESCC tissues and normal paracancerous tissues. A total of 47 genes with differential expression were screened out with *P*-values <0.01 and log2 fold change >2, and the heatmap was plotted (Fig. 1A). We selected KDM5B, which showed the greatest variation, as the study subject. Pan-cancer analysis of KDM5B expression was performed in The Cancer Genome Atlas (TCGA) database, and we found that KDM5B was highly expressed in ESCC patients (Fig. 1B). RT-qPCR and immunohistochemistry further confirmed the overexpression of KDM5B in 30 ESCC tissues (Fig. 1C and D). By RT-qPCR and Western blot of normal human esophageal epithelial cells HET-1A and ESCC cell lines KYSE-150, ECA-109, TE-10, EC-9706, KDM5B was observed to be significantly overexpressed in all ESCC cell lines (Fig. 1E and F). Overall, KDM5B was overexpressed in ESCC patients and ESCC cells and may be related to the development of ESCC.

**Fig. 2.**
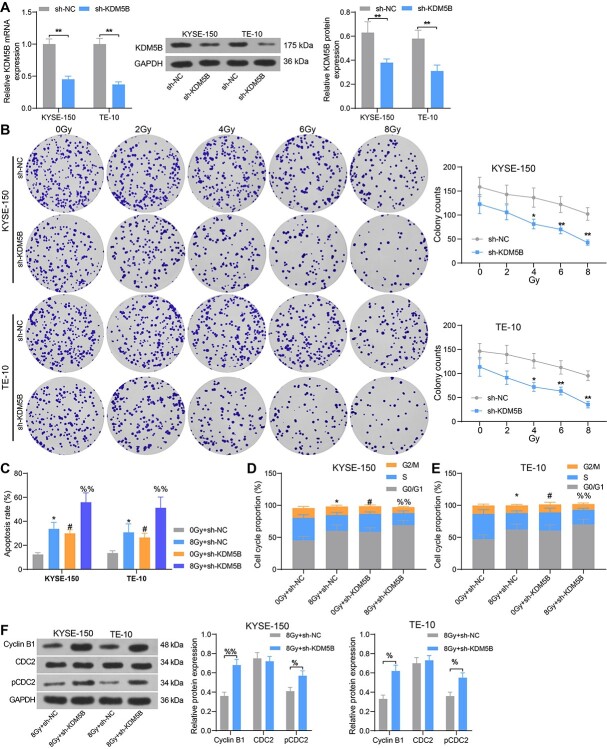
Inhibition of KDM5B promotes sensitivity of ESCC cells to radiotherapy and elevates the apoptosis rate. ESCC cells were transfected with sh-KDM5B or sh-NC. (A) mRNA and protein expression of KDM5B in cells by RT-qPCR and Western blot. (B) Cell colony formation was detected by colony formation after 24 h of radiation treatment. (C) Cell apoptosis assessed using flow cytometry. (D–E) Cell cycle distribution examined using flow cytometry. (F) The expression of cell cycle-related proteins Cyclin B1, CDC2 and pCDC2 tested using Western blot analysis. ^*^P < 0.05, ^*^^*^P < 0.01; #*P* < 0.05 compared with 0 Gy + sh-NC; %*P* < 0.05, %%*P* < 0.01 compared with 8 Gy + sh-NC. The results were measurement data, which were expressed as the mean ± SD. Comparisons between multiple groups analyzed by two-way ANOVA with Tukey’s post hoc test. The experiment was independently repeated three times.

### Inhibition of KDM5B expression promotes sensitivity of ESCC cells to radiotherapy and apoptosis rate

The promoting effects of KDM5B on radiation sensitivity have been validated in oral squamous cell carcinoma [12]. To investigate whether KDM5B has an effect on the sensitivity of ESCC cells to radiotherapy, we knocked down KDM5B in ESCC cells KYSE-150 and TE-10. The results of RT-qPCR and Western blot verified the successful transfection (Fig. 2A). The cells were subjected to different doses (0, 2, 4, 6 and 8 Gy) of radiation, and cell viability was assessed by colony formation assays. The results showed that inhibition of KDM5B expression significantly augmented the sensitivity of KYSE-150 and TE-10 cells to radiation, and the downregulation of cell viability under 8 Gy radiation was more pronounced (Fig. 2B). We then selected cells treated with 8 Gy of radiation for 24 h for following assays (with those irradiated with 0 Gy as control). The cell apoptosis and cycle distribution were assessed by flow cytometry. In KYSE-150 and TE-10 cells, radiation at 8 Gy increased apoptosis, while knockdown of KDM5B increased the sensitivity of cells to radiation and again significantly increased apoptosis (Fig. 2C). Meanwhile, we found KYSE-150 and TE-10 cells under 8 Gy irradiation showed an increase in G1 phase cells and a significant decrease in S phase cells. KDM5B depletion resulted in enhanced cell sensitivity and increased cycle arrest after radiation (Fig. 2D and E). Western blot showed that the protein expression of Cyclin B1 and pCDC2 was significantly enhanced in KYSE-150 and TE-10 cells transfected with sh-KDM5B (Fig. 2F). These findings suggest that inhibition of KDM5B sensitizes ESCC cells to radiotherapy and promotes apoptosis.

### Inhibition of KDM5B activates PIK3C3 expression by promoting H3K4me3 methylation of the PIK3C3 promoter

**Fig. 3.**
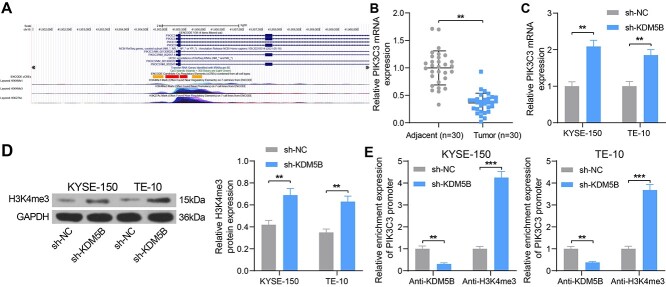
Inhibition of KDM5B activates PIK3C3 expression by promoting H3K4me3 methylation modification of the PIK3C3 promoter. (A) UCSC prediction of PIK3C3 histone methylation activation. (B) Detection of PIK3C3 expression in ESCC tissues by RT-qPCR. (C) Detection of mRNA expression of PIK3C3 in cells after transfection by RT-qPCR. (D) The protein expression of H3K4me3 in cells after transfection using Western blot. (E) Detection of PIK3C3 promoter enrichment by Anti-KDM5B and Anti-H3K4me3 examined using ChIP-qPCR. ^*^^*^P < 0.01, ^*^^*^^*^P < 0.01 compared with adjacent tissues or sh-NC. The results were measurement data, which were expressed as the mean ± SD (n = 30). Comparisons between two groups were conducted using paired t-test (B), and comparisons between multiple groups analyzed by two-way ANOVA (C, D, E) with Tukey’s post hoc test. The experiment was independently repeated three times.

In our previous study, PIK3C3 was found to be poorly expressed in ESCC, acting as a tumor suppressor [7]. The UCSC database (http://genome-asia.ucsc.edu/) demonstrated the presence of the H3K4me3 activation in the PIK3C3 promoter (Fig. 3A), indicating that PIK3C3 may be regulated by the histone demethylase KDM5B. The RT-qPCR assay revealed a significant reduction in PIK3C3 expression in ESCC sample tissues (Fig. 3B). In KYSE-150 and TE-10 cells with knockdown of KDM5B, the mRNA and protein expression of PIK3C3 was significantly increased (Fig. 3C and D). Subsequently, ChIP-qPCR assay confirmed that the enrichment of PIK3C3 promoter by KDM5B was reduced and the enrichment of PIK3C3 promoter by H3K4me3 was much restored after inhibition of KDM5B expression (Fig. 3E). It indicated that inhibition of KDM5B activated PIK3C3 expression by promoting H3K4me3 methylation modification of the PIK3C3 promoter.

### Overexpression of PIK3C3 promotes ESCC cell sensitivity to radiotherapy and apoptosis

**Fig. 4.**
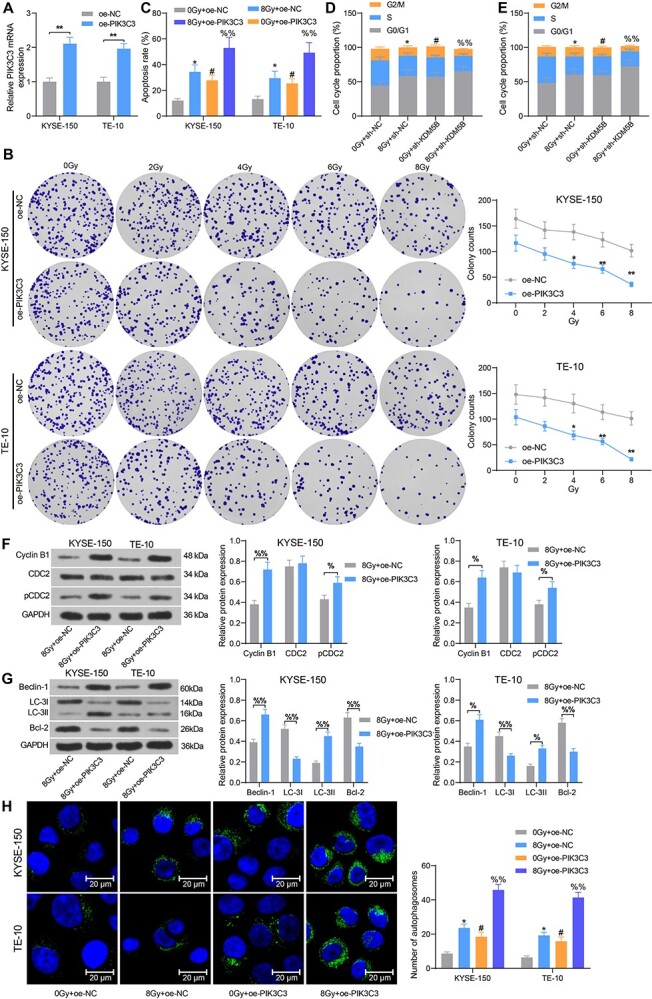
Overexpression of PIK3C3 promotes cell sensitivity to radiotherapy and apoptosis in ESCC cells. ESCC cells were transfected with oe-PIK3C3 or oe-NC. (A) Detection of mRNA expression of PIK3C3 in cells by RT-qPCR. (B) Cell colony formation was detected by colony formation assay after 24 h of radiation treatment. (C) Cell apoptosis measured using flow cytometry. (D–E) Cell cycle distribution examined using flow cytometry. (F) The expression of cell cycle-related proteins Cyclin B1, CDC2 and pCDC2 tested using Western blot analysis. (G) The expression of autophagy- and apoptosis-related proteins Beclin-1, LC-3I, LC-3II and Bcl-2 tested using Western blot analysis. (H) The number of autophagosomes in cells examined using MDC staining. ^*^P < 0.05, ^*^^*^P < 0.01; #*P* < 0.05 compared with oe-NC or 0 Gy + oe-NC; %*P* < 0.05, %%*P* < 0.01 compared with 8 Gy + oe-NC. The results were measurement data, which were expressed as the mean ± SD. Comparisons between multiple groups analyzed by two-way ANOVA with Tukey’s post hoc test. The experiment was independently repeated three times.

First, we overexpressed PIK3C3 in ESCC cells KYSE-150 and TE-10. RT-qPCR assay presented that the mRNA expression of PIK3C3 was significantly elevated after the oe-PIK3C3 transfection (Fig. 4A). After treating each group of cells with 0, 2, 4, 6, and 8 Gy of radiation for 24 h, it was observed that the oe-PIK3C3 significantly increased the sensitivity of the cells to radiotherapy (Fig. 4B). Flow cytometry results exhibited that overexpression of PIK3C3 significantly increased the sensitivity of cells to radiation, contributing to enhanced apoptosis (Fig. 4C) and suppressed cell cycle entry (Fig. 4D and E). Consistently, the protein expression of Cyclin B1 and pCDC2 was significantly boosted in KYSE-150 and TE-10 cells after PIK3C3 upregulation (Fig. 4F).

PIK3C3 has been shown to play an imperative role in the autophagy of cancer cells [13]. The expression of Beclin-1 and LC-3II was elevated, whereas LC-3I expression, LC-3II/I ratio, and Bcl-2 was significantly declined after overexpression of PIK3C3, as revealed by Western blot (Fig. 4G). MDC staining was conducted to measure the number of autophagosomes in each group of cells after radiation. The results exhibited that the number of autophagosomes was enhanced after radiation, which was further promoted after the oe-PIK3C3 treatment (Fig. 4H). Therefore, overexpression of PIK3C3 in ESCC cells can promote radiosensitivity and apoptosis.

### Knockdown of PIK3C3 attenuates the inhibitory effect of sh-KDM5B on ESCC cell radio-resistance

**Fig. 5.**
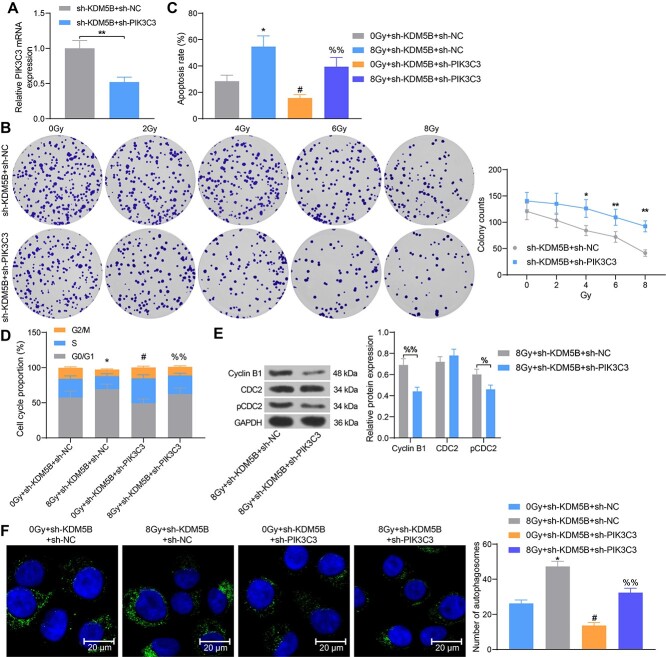
Knockdown of PIK3C3 attenuates the inhibitory effect of sh-KDM5B on ESCC cell radio-resistance. ESCC cells transfected with sh-KDM5B were further transfected with sh-PIK3C3 or sh-NC. (A) Detection of mRNA expression of PIK3C3 in cells by RT-qPCR. (B) Cell colony formation was detected by colony formation after 24 h of radiation treatment. (C) Cell apoptosis measured using flow cytometry. (D) Cell cycle distribution examined using flow cytometry. (E) The expression of cell cycle-related proteins Cyclin B1, CDC2 and pCDC2 tested using Western blot analysis. (F) The number of autophagosomes in cells examined using MDC staining. ^*^P < 0.05, ^*^^*^P < 0.01; #*P* < 0.05 compared with 0 Gy + sh-KDM5B + sh-NC or sh-KDM5B + sh-NC; %%*P* < 0.01 compared with sh-KDM5B + sh-NC or 8 Gy + sh-KDM5B + sh-NC. The results were measurement data, which were expressed as the mean ± SD. Comparisons between two groups were conducted using unpaired t-test (A), and comparisons between multiple groups analyzed by one-way (C, F) or two-way ANOVA (B, D, E) with Tukey’s post hoc test. The experiment was independently repeated three times.

To further investigate whether the KDM5B/PIK3C3 axis involved in ESCC cell sensitivity to radiotherapy, we selected KYSE-150 cells with higher activity as the study subject. Sh-PIK3C3 was further transfected into KYSE-150 cells with knockdown of KDM5B, and it was confirmed that the mRNA expression of PIK3C3 was reduced in sh-KDM5B + sh-PIK3C3-transfected cells relative to the sh-KDM5B + sh-NC group (Fig. 5A). After that, reduced sensitivity of cells to radiotherapy (Fig. 5B), inhibited apoptosis (Fig. 5C), and alleviated G2/M arrest were observed (Fig. 5D). Western blot showed that the expression of Cyclin B1 and pCDC2 at the protein level was significantly reduced in KYSE-150 cells after further low expression of PIK3C3 (Fig. 5E). MDC staining assay revealed that the cells after co-transfection of sh-KDM5B + sh-PIK3C3 contained fewer autophagosomes than the transfection of KDM5B + sh-NC (Fig. 5F). Taken together, knockdown of PIK3C3 attenuates the radiosensitivity of sh-KDM5B to ESCC cells.

### Depletion of KDM5B suppresses the radio-resistance of ESCC in mouse xenografts

**Fig. 6.**
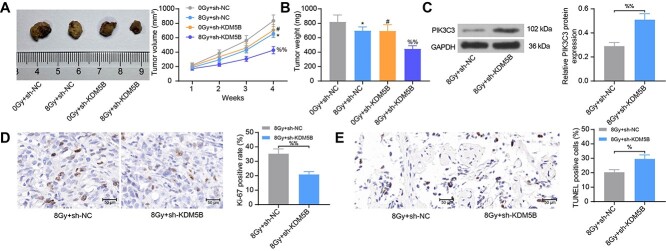
Inhibition of KDM5B inhibits the radio-resistance of ESCC in mouse xenografts. (A) Tumor volume growth of nude mice after radiation. (B) changes in tumor weight. (C) The protein expression of PIK3C3 in tumor tissues examined using Western blot analysis. (D) Immunohistochemical assessment of the percentage of Ki-67-positive cells in tumor tissues. (E) The proportion of apoptotic cells in tumor tissues assessed using TUNEL staining. ^*^P < 0.05; #*P* < 0.05 compared with 0 Gy + sh-NC; %*P* < 0.05, %%*P* < 0.01 compared with 8 Gy + sh-NC. The results were measurement data, which were expressed as the mean ± SD. Comparisons between two groups were conducted using unpaired t-test (C, D, E), and comparisons between multiple groups analyzed by one-way (B) or two-way ANOVA (A) with Tukey’s post hoc test.

To determine whether KDM5B affects the sensitivity of ESCC cells to radiation therapy *in vivo*, we tested the effect of sh-KDM5B on radiation efficacy in BALB/c nude mice carrying xenografts formed by KYSE-150 cells. Radiation was started 7 days after cancer cell inoculation. The results revealed that the tumor volume and weight were significantly reduced by radiation. Silencing of KDM5B resulted in reduced activity of tumor cells *in vivo*, as evidenced by increased sensitivity to radiation (Fig. 6A and B). Tumors with low expression of KDM5B had higher levels of PIK3C3 (Fig. 6C) and reduced Ki-67 staining (Fig. 6D), as revealed by Western blot analysis and immunohistochemical staining, respectively. In addition, TUNEL-positive cells were significantly elevated in tumors with low KDM5B expression than that in controls (Fig. 6E). These observations provide further evidence for the role of KDM5B in regulating the radiosensitivity of ESCC cells to radiation.

## DISCUSSION

As the H3K4me2/3 residue characterizes the transcription initiation site of the transcriptional gene, and demethylation of H3K4me3 is linked to transcriptional repression, making it a possible contributor to the downregulation of tumor suppressors [14]. We previously identified the sensitivity-promoting effects of PIK3C3 on ESCC radiotherapy [7]. However, the specific mechanism of PIK3C3 in radio-resistance of ESCC remains unclear. Here, we uncovered the relevance of KDM5B, a histone demethylase responsible for removing H3K4me2/3 activation marker, to the decline of PIK3C3 expression in ESCC. Importantly, we dissected the molecular mechanisms of this regulation by showing that KDM5B reduces the expression of PIK3C3 via the H3K4me3 demethylation modification at its promoter, thus mediating cell cycle arrest and autophagy, contributing to ESCC cell radio-resistance.

In this study, we conducted mRNA-based microarray analysis to screen differentially expressed genes in ESCC. KDM5B was identified as one of the most significantly overexpressed one, which was consistent with a previous study [15]. In addition, overexpression of KDM5B was related to the histologic type, clinical stages, lymph node metastasis, and poor prognoses of patients with human laryngeal squamous cell carcinoma and non-small cell lung cancer [16,17], indicating its potential diagnostic role. More relevantly, a positive correlation between KDM5B expression and chemotherapy resistance was observed in patients with epithelial ovarian cancer (odds ratio = 36.81, *P* < 0.001) [18]. The supporting effects of KDM5B on chemoresistance have been implicated in gastric cancer [19], melanoma [20], as well as endometrial carcinoma [21]. In addition, silencing of KDM5B blocked oncogenicity, stemness and augmented radiation sensitivity in human oral carcinoma [12]. Moreover, KDM5B knockdown significantly suppressed proliferation and decreased the proportion of cells in S and G2/M phase of prostate cancer cells [22]. Bak protein was upregulated, whereas Bcl-2 was downregulated in the KDM5B-knockdown cells, indicating KDM5B maintained head and neck squamous cell carcinoma survival by regulating Bcl family members [23]. Using canine melanoma cell lines, Tobin *et al.* determined that broad spectrum KDM inhibitors resulted in decreased cell survival and prolonged DNA damage repair kinetics [24]. At the molecular level, we found that silencing KDM5B was sufficient to enhance the expression of Cyclin B1 and pCDC2, cell cycle-related factors, further substantiating the cell cycle regulator role of KDM5B in ESCC. In agreement with our finding, in Kyse150 cells, a more significant elevation in pCDC2 and Cyclin B1 was found in the raltitrexed combined with irradiation group relative to irradiation alone group, along with a G2 phase arrest [25].

Interestingly, KDM5B was found to promote the radio-resistance of non-small cell lung cancer through the decline of PTEN expression [26]. As a consequence, we postulated that KDM5B exerts a similar effect on ESCC cells via regulating the expression of PIK3C3. Moreover, KDM5B^high^ cells showed increased PI3K pathway activation in oral cancers [27]. In ESCC, KDM5B was revealed to bind to the promoters of Th1-type chemokines to alter H3K4 methylation [28]. Also, we elucidated that suppression of KDM5B activated PIK3C3 expression by promoting H3K4me3 methylation modification of the PIK3C3 promoter. Previously, SRSF1 has been indicated to inhibit autophagosome formation by reducing the accumulation of LC3-II and numbers of autophagosomes in lung cancer cells by directly binding to PIK3C3 [29]. Beclin-1 is a major component of the PIK3 complex, which plays an imperative part in membrane trafficking and restructuring involved in autophagy [30]. This study validated that upregulation of PIK3C3 enhanced the protein expression of Cyclin B1, pCDC2, Beclin-1, and LC-3II, while lowered the expression of LC-3I, Bcl-2 and LC-3II/I. Shannan *et al.* displayed that KDM5B favored cell survival by transcriptional modulation of genes related to cell cycle, DNA repair and cell death [31]. Our observations derived from rescue experiments exhibited that silencing of PIK3C3 mitigated the effects of sh-KDM5B on ESCC cell autophagosome formation, cell cycle arrest, and radio-resistance.

## CONCLUSION

The findings of our study support the posit that the KDM5B/PIK3C3 axis is a promising novel regulatory network in the radiotherapy of ESCC. We have provided evidence suggesting that KDM5B reduces the expression of the tumor suppressor PIK3C3 via H3K4me3-mediated demethylation, which ultimately reduces ESCC cell radiosensitivity. However, our research is still a preliminary one, indicating more experiments in this field are required in the future.
